# Investigating the Impact of Synchrotron THz Radiation on the Corneal Hydration Using Synchrotron THz-Far Infrared Beamline

**DOI:** 10.3390/s22218261

**Published:** 2022-10-28

**Authors:** Negin Foroughimehr, Zoltan Vilagosh, Ali Yavari, Andrew Wood

**Affiliations:** 1 School of Health Sciences, Swinburne University of Technology, Melbourne, VIC 3122, Australia; 2 Australian Centre for Electromagnetic Bioeffects Research, Swinburne University of Technology, Melbourne, VIC 3122, Australia; 3 School of Science, Computing and Engineering Technologies, Swinburne University of Technology, Melbourne, VIC 3122, Australia

**Keywords:** terahertz radiation, attenuated total reflection, dosimetry, synchrotron, corneal water content, ophthalmology

## Abstract

Due to increasing interest in imaging, industrial, and the development of wireless communication operating at THz frequencies, it is crucial to ascertain possible health impacts arising from exposure to THz radiation. This paper reports on the pilot study of transmittance and absorbance spectra of the porcine cornea following THz frequency irradiation at a synchrotron THz/Far-IR beamline. The exposure period was 4 hours. One cornea was exposed to the radiation, with a second cornea acting as a control. An Attenuated Total Reflection (ATR) apparatus was used, and the frequency range of 2.4 to 8 THz was selected to evaluate any changes. It was found that the synchrotron THz radiation intensities are too low to produce induced corneal injury, but may lead to subtle changes in the state of water. Our results suggest that THz spectroscopy is a promising modality for corneal tissue hydration sensing.

## 1. Introduction

High Gigahertz (GHz) and Terahertz (THz) frequencies are becoming important in the communication, security, and imaging fields. The Infrared (IR) region is a wide range, which is divided into three regions: Near-IR, Mid-IR, and Far-IR. The Near-IR ranges from 800 to 2500 nm, which corresponds to the wavenumber range of 12,500 ${\mathrm{cm}}^{-1}$ to 4000 ${\mathrm{cm}}^{-1}$. The Mid-IR region (120–12 THz) is where that enables observation of the fundamental bands of molecular vibrations. The wavelength and wavenumber ranges of the Mid-IR are 2.5–25 μm, and 4000–400 ${\mathrm{cm}}^{-1}$, respectively.

The Far-IR and THz frequency range overlap is generally accepted as being between the IR and microwave regions of the Electromagnetic (EM) spectrum, corresponding to wavelengths of 3 to 0.033 mm, with frequencies of 100 GHz to 10 THz, (wavenumber range from 3.3 ${\mathrm{cm}}^{-1}$ to 333 ${\mathrm{cm}}^{-1}$; The energy range of the THz band is from 4.1 × 10${}^{-4}$ to 4.1 × 10${}^{-3}$ eV [1,2,3].

Given that the THz band has a huge potential for its usage in future 6G communication devices, researchers have started exploring the 0.1–10 THz band [4]. Two atmospheric spectral windows in the lower part of the THz band centred at 300 GHz and 350 GHz offer 47 GHz of continuous bandwidth with low atmospheric attenuation.

Moreover, in recent years, THz systems have emerged as a unique non-contact and non-invasive imaging technique in biomedical fields [5]. THz radiation indicates high sensitivity to water, thus making it easier to distinguish tissues with different water content using THz-based techniques. [6]. For instance, cancerous tissues can be distinguished from healthy tissues since tumour tissues contain more water than normal tissues due to a higher level of metabolism [7]. The potential application of THz sensing in the field of ophthalmology has been reported by other researchers [8,9]. Bennett et al., reported on the use of both THz imaging and spectroscopy systems [10]. Moreover, Singh et al., developed a pulsed reflective THz imaging system specifically for hydration sensing [11].

Since the penetration depth of THz frequencies in human tissue is around 1 mm or less, any adverse effects would be expected to be observed in the skin and the eyes. We have previously reported on a computational set up to study the EM radiation’s biological impacts on the cornea [12]. At present, there is sparse experimental research regarding the impact of THz radiation exposure on any biological tissue including the cornea. The previous studies involved THz sensing of corneal tissue-induced damages and have used various THz radiation sources and hydration measurement systems. For instance, Iomdina et al., reported on the study of transmittance and reflectance of the cornea and sclera in the range of 0.13 to 0.32 THz followed by exposure to a backward-wave oscillator (BWO) with an effective bandwidth frequency of 10 MHz [13]. In another study, they explored the THz scanning of hydration of corneal tissue followed by damage caused by B-band ultraviolet (UVB) exposure at 50 Hz [14], while some studies have investigated THz sensing of ocular tissues, no studies have previously reported on the use of synchrotron continuous THz radiation source for both exposing and evaluating biological changes in corneal tissues or other ocular tissues. Furthermore, the mentioned studies do not contain the frequencies under consideration for this paper.

The significance of this research is that it highlights the possibility of using synchrotron THz irradiation as a THz source as an irradiation mechanism for sample exposure, and as a spectroscopic analysis tool to study the biological impacts of the THz band, where there is limited access to THz emitters.

A pilot study to investigate the impact of THz radiation, including water balance variation of corneal tissues, following continuous synchrotron THz exposure was conducted at the Australian Synchrotron THz beamline. The purpose of the study was to establish the utility of the THz beamline for exposure studies.

We previously used synchrotron radiation to estimate the refractive index of homogeneous samples in the 1.0 to 4.0 THz range [15], as well as water-based substances in the 2.0 THz region [16]. The purpose of this work was to identify the threshold value of corneal-induced injury using a continuous synchrotron THz radiation source. We also employed our previously used technique to evaluate the water balance of exposed corneal tissues. The use of synchrotron radiation to expose tissues to radiation is a novel approach. Synchrotron radiation is continuous and stable over many hours giving a THz exposure not available with other THz sources such as quantum cascade lasers.

### 1.1. The Physiology of the Cornea

The cornea is the anterior, transparent region of the eyeball and is known as the major refracting element of the eye, which separates the air with a refractive index of 1.00 from the aqueous humour with a refractive index of 1.33 [17,18]. The cornea is elliptical in shape and the curvature being steeper in the centre [19]. The thickness of the central region is 0.52mm with a standard deviation of 0.04 mm. The cornea is thicker at the periphery where its thickness value is about 0.65 mm [20]. In contrast, many animals’ corneas have the same thickness at both central and peripheral regions [21].

The relative ocular dimensions of some commonly studies species are listed in Table 1.

The major substance in living systems is liquid water averaging about 75% [22]. The human cornea contains 75–80% water. The remaining 20–25% of the cornea consists of collagen with soluble proteins and polysaccharides [23]. The cornea consists of five layers, from anterior to posterior the layers are epithelium, bowman’s layer, stroma, Descemet’s membrane, and endothelium. The epithelium layer is the external surface of the cornea, which is avascular but contains nerve endings [24] that make the cornea highly sensitive to pain [25]. The epithelium is five to seven cell layers thick with an overall thickness of 50 μm that remain constant throughout life in the central cornea [26].

Just deep to the epithelium is the Bowman’s layer, which is comprised of two specific collagen types. Its thickness varies from 11 to 17 μm [26]. Collagen is the most common protein present in mammals, allowing cell-matrix and providing tensile strength. The highly ordered hierarchical architecture of collagen provides biological materials with remarkable properties (e.g., mechanical strength, anisotropy, etc.). To date, 28 various types of collagen have been identified, classified as fibrillar and non-fibrillar in forming fibrillar structures depending on the type of chain, bonding, and so on [27,28].

The stroma forms 90% of the total corneal thickness which maintains the corneal shape and strength [20]. Its primarily made up water (78%) and collagen (16%) [25]. Each layer of the stroma is called lamellae and each lamellae is made up of collagen fibrils mainly of type I collagen, with smaller amounts of type III, V, and VI. The collagen fibrils are 20 times smaller than the wavelength of visible light.

The surfaces of the sclera and cornea are covered with a very thin film of tear fluid with a thickness of 10 μm which is most often described as a three-layer film. This multi layered fluid structure has both nutritional and protective properties which establishes itself rapidly after each blink [29]. Although the synchrotron THz radiation intensity is expected to be too low to induce protein denaturation impacts, there is evidence that lower intensities are sufficient to alter water structure [30].

### 1.2. Water Content Evaluation

Stable water content is essential to maintain the cornea in a normal state; a 10% change in water content results in pathological conditions [13]. Two natural states of water in biological tissues are known as free “bulk” water (i.e., freely moving water molecules) and bound water [31] which is associated with tissue molecular components and is not movable. As the name implies, free “bulk” water can move freely within the cell whereas bound water is immobilised by hydrogen bonds in more subtle molecular contact with cellular constituents. The dielectric constant of corneal tissue is determined by three main constituents: collagen fibres, free water, and bound water [32]. The reduced freedom of movement of bound water is equivalent to reducing the temperature of free water, and the dielectric properties change in the same way. The bound water is converted into free water when strong stimulation is applied to the tissue [32]. At THz frequencies, the result is that bound water has a lower absorption coefficient and a higher refractive index when compared to free water at the same temperature [31]. Water illustrates high absorption and reflection coefficient values in the 0.3 to 3 THz frequency range [13]. Moreover, the THz radiation is sensitive to bound and free water content in tissues [32]. Therefore, any slight changes in the water balance of corneal tissues can be detected by obtaining the absorbance spectra of the tissue in this range of frequency. The dielectric properties of the corneal tissue are largely determined by its water content, thus changes in tissue water content utilised as a contrast mechanism in THz imaging [33].

### 1.3. Attenuated Total Reflection Spectroscopy

**Figure 1:**
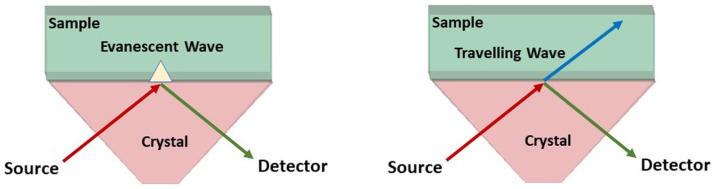
The schematic representation of the ATR apparatus. (**Left**) “True ATR” mode, where the incident radiation is totally reflected at the sample/crystal interface. (**Right**) Partial reflection/partial transmission mode, where some of the signal is transmitted into the sample as a travelling wave.

We have devised a number of innovative approaches to interrogate biological samples with attenuated total reflection (ATR) apparatus (shown in Figure 1) at THz frequencies at THz/Far-IR beamline in the Australian Synchrotron [34]. During total internal reflection, an evanescent wave is generated in the sample. The penetration depth $D_{p}$ of the evanescent wave can be calculated using Equation (1).

(1) $$D_{p}=\frac{\lambda }{2\pi n_{1}\sqrt{Sin^{2}\theta }-{\left(\frac{n_{2}}{n_{1}}\right)}^{2}}$$

where λ is the wavelength of incident radiation in free space, $n_{1}$ is the refractive index of the crystal, $n_{2}$ refractive index of the sample, and θ is the angle of incidence of the radiation. At THz frequency range, the penetration depth into biological tissues is in the order of 0.1 to 0.5 mm [34]. The equation returns a real number $D_{p}$ if the term $sin^{2}\theta$ is larger than ($n_{2}/n_{1}$)${}^{2}$ since the square root of a negative number can only be expressed as a complex number. The Australian Synchrotron THz ATR has a θ = 45${}^{\circ }$ incoming beam angle and is equipped with a diamond crystal. The refractive index (*n*) of the diamond crystal remains stable at 2.4 throughout the THz range whist the n water, and high-water content tissues such as the cornea, reduces from 2.25 at 0.5 THz to ~1.5 at 10 THz [27] According to Equation (1), if θ = 45${}^{\circ }$$sin^{2}\theta$ becomes smaller than ($n_{2}/n_{1}$)${}^{2}$ at $n_{2}=1.7$. The *n* of water is ~1.7 at 5.25 THz, and this is the boundary between total ATR reflection ($n_{water}<$ 1.7 ) and partial reflection/partial transmission ($n_{water}>$ 1.7 ). Thus, if $n_{water}>$ 1.7, at less than 5.0 THz, the ATR apparatus is acting in a partial reflection/partial transmission mode [16], with true ATR at frequencies above 5 THz.

## 2. Methodology

To investigate the potential damage, we used the THz/Far-IR beamline at the Australian Synchrotron, Melbourne, Australia to expose the cornea and evaluate the corneal hydration. The processes of tissue exposure and water content analysis are described in the following sections.

### 2.1. Sample Preparation

**Figure 2:**
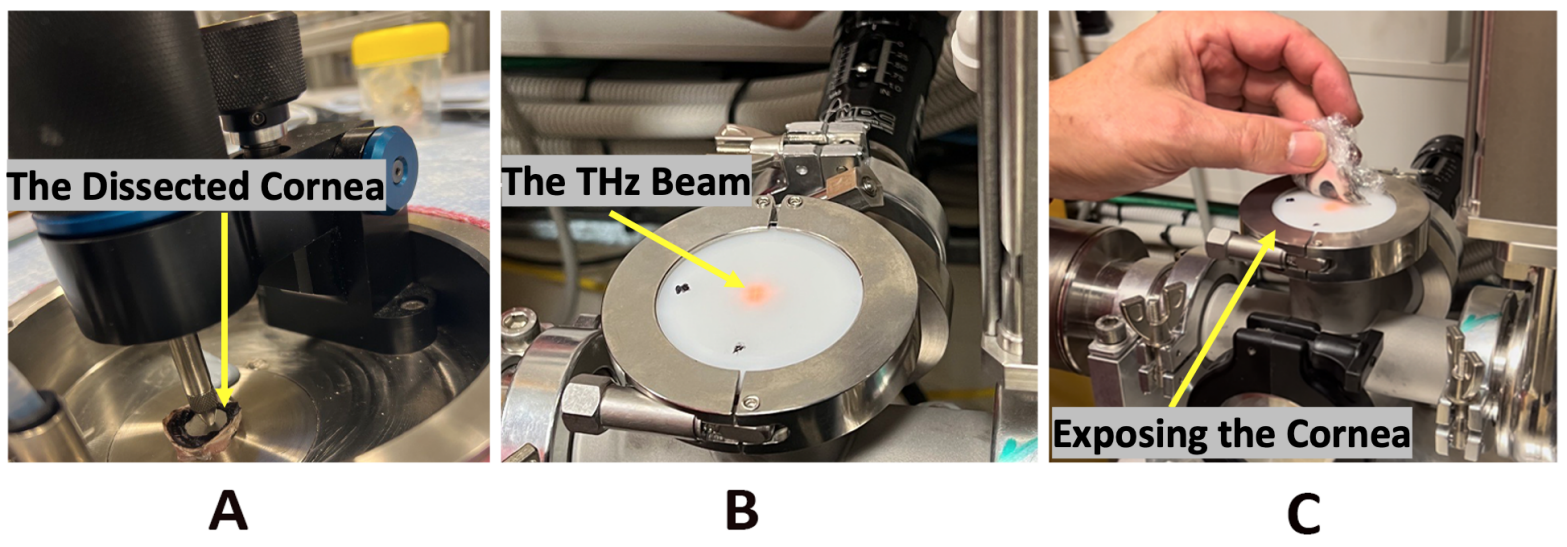
The illustration of the experimental setup (**A**) The examination of the porcine cornea immediately after dissection and before THz exposure using the ATR system (**B**,**C**) the THz/Far-IR beamline at the Australian Synchrotron was used as a radiation source to irradiate the samples. The THz beam is invisible, and by the interaction of the beam with the Teflon plastic, a patch of red light is produced. (The beam is combined of THz/Far-IR and the interaction of the IR portion with the Teflon excites into the red region).

Due to the similarity of porcine and human eye dimensions and ease of access, we used porcine cornea as a surrogate for human ocular tissues. The porcine corneas were sourced from a commercial abattoir, collected before any heat rendering of the carcass, and used on the day of collection. The whole porcine eyes were transported in a cooled container, wrapped in a saline dampened gauze ensured they did not dry out or become waterlogged. The anterior segment of the eyes (i.e., corneas) was dissected using a surgical knife. Prior to one cornea was used for the exposure, the second was a control. The control sample underwent identical handling and was placed to the side of the beam exposure port to maintain an identical temperature to that of the exposed cornea. Care was taken to maintain the physiological hydration levels of the samples by placing the samples in the cling wrap, as shown in Figure 2C. The second pair of corneal samples were placed in saline to evaluate the changes such storage produced over time.

### 2.2. THz Exposure Using the Synchrotron Light Source

We employed the synchrotron THz/Far-IR beamline in the range of 0.5 to 20 THz with a total incident power of approximately 80 μW as a continuous radiation source. In practice most of the incident power density was found to be between 2.3 and 8.9 THz, thus the frequencies outside these boundaries were not considered. The tissues were placed on the radiation source at the focus of the beam, which is illustrated in Figure 2B,C. The tissue was exposed to the synchrotron THz radiation for 4 hours. The size of the radiation spot was 9 mm in diameter (at half maximum), thus intensity is approximately 1.26 $W/m^{2}$.

### 2.3. Data Collection Using the Synchrotron ATR Apparatus

**Figure 3:**
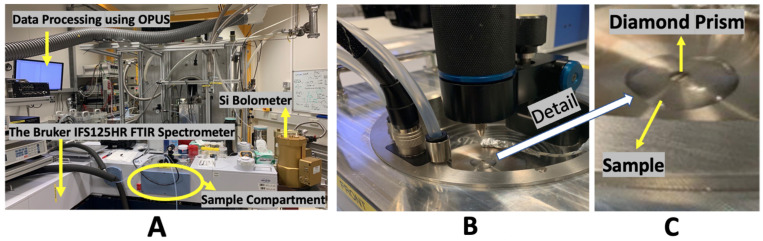
(**A**) The ATR apparatus setup available at the THz/Far-IR Beamline at the Australian Synchrotron in Melbourne, Australia, the ATR is equipped with a Bruker IFS125HR spectrometer with a scanning length of 5 m (**B**) Measuring the reflectance spectra of a sample using the ATR apparatus, which has a diamond prism (n=2.40), and a fixed 45${}^{\circ }$ incoming beam angle that is located below the central window. The sample is placed above this window. (**C**) The close snapshot of the ATR apparatus showing the diamond prism. As noted in Figure 1, the incident radiation is totally reflected at the sample/diamond interface in the true ATR mode, and in Partial reflection/partial transmission mode, the beam is partially absorbed by the transition through the sample and partially reflected and reverted to a travelling wave at the return to the sample/diamond interface.

The data were collected in the THz/Far-IR Beamline at the Australian Synchrotron (Clayton, Victoria). The THz/Far-IR Beamline, and associated spectrometer (Figure 3A,B) were equipped with an ATR apparatus and a diamond prism (refractive index of 2.40, 45${}^{\circ }$ incoming beam angle). Air and water were used to calibrate the ATR apparatus.

The reflectance scans between 2.4 and 8 THz were obtained using a Bruker IFS 125HR Fourier Transform Infrared spectrometer with a Si Bolometer in the range of 10–650 ${\mathrm{cm}}^{-1}$ (Bruker Optics, Ettlingen, Germany). Data were collected with each datapoint being an average of 20 scans. OPUS 8.0 software was utilised for the initial data analysis (Figure 3A). Due to the brightness of the Australian Synchrotron THz source, the ATR apparatus can produce a high-quality scan every 0.3–0.4 s at THz frequencies. The typical signal-to-noise ratio (S/N) ratio is ∼20 times higher as compared with Global sources widely used in table-top Fourier transform (FTIR)-IR spectrometers.

After calibration with pure water (as shown in Figure 3C, for water content evaluation, both corneas were examined prior to exposure using the ATR system (Figure 2A). After exposure to THz radiation of 4 hours, the exposed cornea was examined alongside the control cornea.

### 2.4. Data Analysis

**Figure 4:**
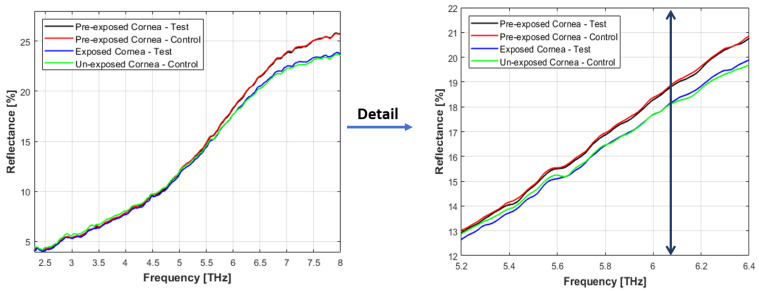
(**Left**) Reflectance data for porcine exposed and unexposed corneal tissues in the THz range (2.4 to 8 THz) corresponding to (79.92 to 266.4 ${\mathrm{cm}}^{-1}$ wavenumber). The data were normalised to air = 100%. Black and red curves were obtained immediately after dissection and the samples not being exposed to THz radiation. Blue and green curves were obtained after four hours of exposure to THz radiation. Exposed sample-test (in blue), and the 4 hours unexposed cornea-control (in green), the same time as the exposed cornea (**Right**) the detail of reflectance spectrum of exposed and unexposed samples.

Reflectance data were collected between the range of 2.4 to 8 THz since the data 158 proved to be reliable between this range, with detector reproducibility decreasing at both 159 Figure 4. Left) Reflectance data for porcine exposed and unexposed corneal tissues in the THz range boundaries of this interval. The obtained data were analysed using Excel and MATLAB. 160 The raw reflectance data were normalised to air reflection = 100% for the reduction of the 161 reflectance due to reflections at the crystal/sample interface [16].

## 3. Results and Discussion

**Figure 5:**
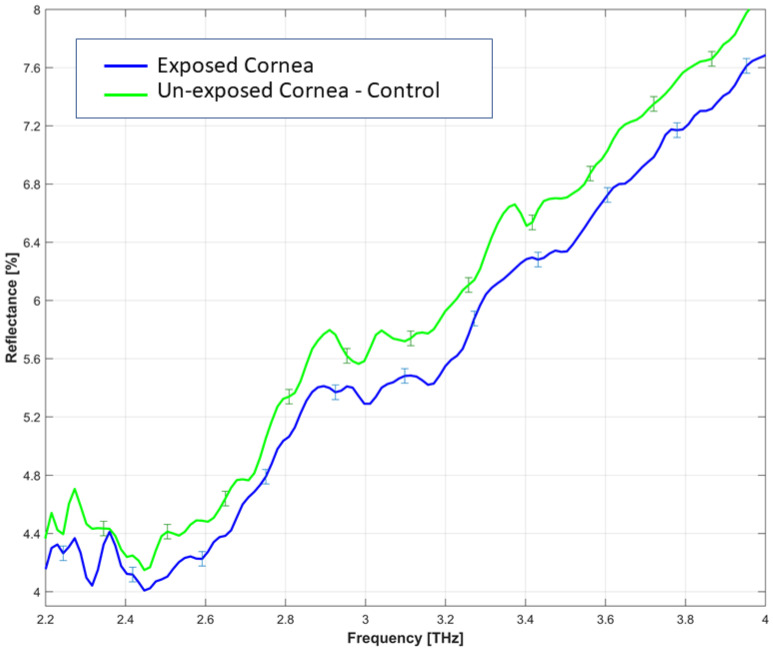
The reflectance spectrum of the exposed sample-test and the 4 hours unexposed cornea control was measured at partial reflection/partial transmission region below 5.0 THz. The data were normalised to air = 100% and represents a reduction in bound water of the exposed sample. The represented error bars of 0.05 are the standard deviation of measured reflectance data of exposed and unexposed samples, each obtained from 20 scans.

The measured reflectance spectrum of the porcine cornea (exposed and unexposed samples) in the frequency range of 2.4 to 8 THz is shown in Figure 4. This includes the “true ATR” reflectance region above 5.0 THz (shown in the right panel of Figure 4) and the partial reflection/partial transmission region below 5.0 THz (shown in Figure 5).

The reflectance spectral scans of pre-exposed corneal tissues have a near-similar form. However, reflectance data obtained after 4 hours of exposure dropped from 25.72% and to 23.64% and at 8 THz for exposed and control corneas, respectively. Similarly, the measured reflectivity of the control sample decreased from 25.74% to 23.52% for the unexposed control sample after four hours.

Figure 4 illustrates the decrease in bound water over time in both corneas. It represents decrease in reflectance over time at >5.0 THz, indicating a transition from bound water to bulk water. The analysis of the reflectance spectrum revealed that the reflectance value of the cornea was not different when comparing the exposed sample with the control at above 5 THz. As shown in the detail of Figure 4 (right panel), the reflectance value of the unexposed control cornea starts to slightly drop above 6.09 THz when compared to the exposed sample.

**Figure 6:**
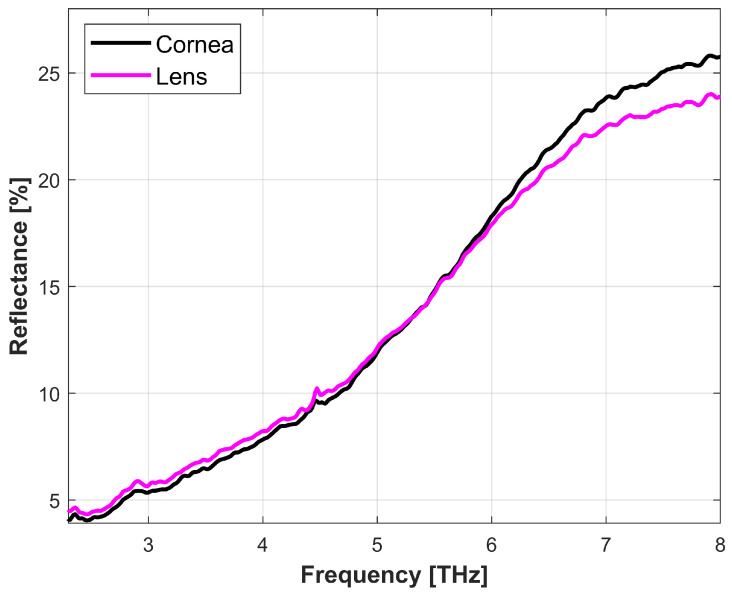
The reflectance spectra of porcine cornea and lens in the range of 2.4 to 8 THz.

To validate the sensitivity of the proposed method, we also examined the lens to assess its reflectance spectrum. The average water content of the human lens is approximately about 64–69% [35]. The data in Figure 6 reports the reflectivity of the porcine cornea and lens. This measurement was taken immediately upon dissection of the tissues. The reflectance spectra of the lens and cornea clearly show that the cornea water content dominates radiation reflection, confirming that the interaction between THz radiation range and porcine corneal tissue is dominated by water.

The preliminary results indicate that using the ATR system, based on the use of a continuous synchrotron THz radiation source, could be a unique method for scanning hydration in biological samples. We have also found that the available synchrotron generated THz intensities are too low to produce any discernible degradation in the corneal structure. However, it was found that the rate of release-bound water is increased in the presence of synchrotron THz radiation.

We are presently devising some methods to produce higher intensities at GHz frequencies and using the synchrotron to examine the impacts on the THz spectra, to discern changes in absorption bands associated with protein structure.

It should be noted that using differences in radiation sources (i.e., various THz frequencies), the exposure time, and the state of the biological sample could lead to different threshold values of THz radiation energy parameters, while the results are encouraging, we will need to quantify the best frequency of the THz radiation source at which to produce induced corneal injuries.

In conclusion, we evaluated the biological impacts of the THz region on the cornea (in terms of hydration variation), following excessive THz exposure. The ATR apparatus at the Australian Synchrotron provides rapid acquisition of absorbance and transmittance data of tissues with minimum sample preparation. This provides an appropriate environment to study the effects of EM radiation on biological tissues, and to establish the use of the ATR technique for THz scanning in ophthalmology.

## 4. Conclusions

THz radiation band is becoming desirable for communication systems as well as THz scanning imaging systems. Currently, the biological impacts of THz waves during irradiation are a cause of concern. In this study, a novel and potentially very sensitive method has been demonstrated to evaluate the biological impacts of THz radiation on corneal tissue in terms of hydration variation, following excessive synchrotron THz exposure. Reflectance spectral scans over the range of 2.4 to 8 THz (79.9 to 266.4 ${\mathrm{cm}}^{-1}$ wavenumber) were performed using the ATR apparatus available at the Australian Synchrotron. The advantage of using a bright synchrotron THz radiation source is the speed with which the spectroscopic data can be obtained in a short time frame. The results provide the basis for future research work on the development of producing higher intensities and using the synchrotron in association with THz-ATR to examine the biological impacts and the development of THz communication systems, as well as THz sensing systems in ophthalmology.

## Figures and Tables

**Table 1:** Average dimensions of the cornea in different species [21].

Species	Size of Eyeball (mm)	Corneal Diameter (mm)	Corneal Thickness (mm)	Curvature (mm)
Human	24	11	0.52	7.9
Rabbit	17	12	0.4	8
Rat	6	5.5	0.2	3
Mouse	3.5	3	0.15	1.75
Porcine	20	15	0.7	10
Ox	35	20	0.8	17.5
Cynomolgus	18	9	0.4	7
